# Transcriptomic and functional analyses reveal an antiviral role of autophagy during pepper mild mottle virus infection

**DOI:** 10.1186/s12870-020-02711-x

**Published:** 2020-10-29

**Authors:** Yubing Jiao, Mengnan An, Xiaodong Li, Man Yu, Xiuxiang Zhao, Zihao Xia, Yuanhua Wu

**Affiliations:** 1 College of Plant Protection, Shenyang Agricultural University, Shenyang, 110866 China; 2 General Station of Forest and Grassland Pest and Diseases Control, National Forestry and Grassland Administration, Shenyang, 110034 China

**Keywords:** Pepper mild mottle virus (PMMoV), Pepper, Transcriptomic analysis, Autophagy, *Nicotiana benthamiana*, Defense response

## Abstract

**Background:**

Pepper mild mottle virus (PMMoV) is a member in the genus *Tobamovirus* and infects mainly solanaceous plants. However, the mechanism of virus-host interactions remains unclear. To explore the responses of pepper plants to PMMoV infection, we analyzed the transcriptomic changes in pepper plants after PMMoV infection using a high-throughput RNA sequencing approach and explored the roles of host autophagy in regulating PMMoV infection.

**Results:**

A total of 197 differentially expressed genes (DEGs) were obtained after PMMoV infection, including 172 significantly up-regulated genes and 25 down-regulated genes. The Gene Ontology (GO) and Kyoto Encyclopedia of Genes and Genomes (KEGG) pathway analyses revealed that most up-regulated DEGs were involved in plant abiotic and biotic stresses. Further analyses showed the expressions of multiple *autophagy-related genes* (*ATG*s) were increased after PMMoV infection in pepper and *Nicotiana benthamiana* plants. Through confocal microscopy and transmission electron microscopy, we have found that PMMoV infection in plant can induce autophagy, evidenced by the increased number of GFP-ATG8a fluorescent punctate and the appearance of double membrane autophagic structures in cells of *N. benthamiana*. Additionally, inhibition of autophagy significantly increased PMMoV RNA accumulation and aggravated systemic PMMoV symptoms through autophagy inhibitor (3-MA and E64d) treatment and silencing of *NbATG* expressions by a *Tobacco rattle virus*-induced gene silencing assays. These results indicated that autophagy played a positive role in plant resistance to PMMoV infection.

**Conclusions:**

Taken together, our results provide a transcriptomic insight into pepper responding to PMMoV infection and reveal that autophagy induced by PMMoV infection has an antiviral role in regulating PMMoV infection. These results also help us to better understand the mechanism controlling PMMoV infection in plants and to develop better strategies for breeding projects for virus-resistant crops.

**Supplementary Information:**

**Supplementary information** accompanies this paper at 10.1186/s12870-020-02711-x.

## Background

Pepper (*Capsicum annuum* L.) is a solanaceous vegetable crop with a great economic value worldwide [1]. Pepper is known to be vulnerable to fungal, bacterial and viral pathogens. For example, virus infection can cause serious pepper yield and quality reductions under both field and protective cultivation conditions [2]. To date, about 13 plant viruses have been reported to infect pepper crops worldwide [3, 4]. Among these known plant viruses, pepper mild mottle virus (PMMoV) is a great threat to pepper production, due mainly to its high contagious nature and long persistence in soil [5].

PMMoV is a positive-sense single-stranded RNA (+ssRNA) virus in the genus *Tobamovirus* of the family *Virgaviridae* [6, 7]. The genome of PMMoV, which contains a m7GpppG cap structure at 5′ end and its 3′ end could be folded into tRNA structure, consists of 6356–6357 nucleotides (nts) and encodes at least four proteins: 126-kDa (p126) and 183-kDa (p183) replication-associated proteins sharing the same initiation codon, movement protein (MP) and coat protein (CP) [6]. PMMoV infection initially causes mild foliar mosaic symptoms followed by mottling and malformation of leaves and fruits, resulting in significant losses of pepper yield and cash values [8]. Moreover, PMMoV infection cause some human clinical illness such as fever, abdominal pains and pruritus [9]. This study was designed to identify host factor(s) that regulate PMMoV infection in pepper and can be utilized to develop effective PMMoV control strategies.

Viral infection in plant is a sophisticated process that involves many un-elucidated interactions between virus and host plant. It was reported that during virus initial invasion, host plant can activate signaling cascades to recognize the invading virus, and during virus infection, the host can alter the expressions of various metabolism-, signal transduction- and/or protein synthesis-related genes to suppress virus further replication and spread [10]. We speculated that identifications of host factor(s) that can regulate host-pathogen interactions should provide useful insights into the mechanism controlling host defense responses to virus infection, and important molecular markers for future breeding projects of disease resistance [11]. Because host-virus interaction is complex and involves many physiological processes, transcriptomic analysis has become increasingly important for this type of study [12]. RNA sequencing (RNA-seq) is a powerful technique for the identifications of genes whose expressions can be altered during abiotic and biotic stresses. For example, RNA-seq has been used to study global gene expression profiles and signal transduction pathways involved in plant defense responses to various stresses, including virus infection [13–15]. Genome of pepper has recently been fully sequenced [16, 17]. This valuable resource allows us to investigate transcriptomic changes during PMMoV infection in pepper plant.

Autophagy is an evolutionarily conserved intracellular degradation pathway in eukaryotes. It can transport cytoplasmic components, including proteins and dysfunctional organelles, to lysosomes (in animals) or vacuoles (in yeast and plants) for degradation [18, 19]. Autophagy has been reported to stabilize cells and organs, to regulate programmed cell death (PCD) [20], and to control plant development, regeneration, senescence, other biological processes, and responses to abiotic stresses [21]. Autophagy can also cause hypersensitive response (HR)-mediated cell death during initial plant responses to pathogens, and limit the spread of PCD beyond the initial infection site [22]. Moreover, autophagy is considered to participate in plant defense against virus infection through different mechanisms. For example, Neighbor of BRCA1 gene 1 (NBR1)-mediated selective autophagy pathway can inhibit cauliflower mosaic virus (CaMV) infection via binding to viral capsid protein and virus particles [23]. Autophagy can target the virulence factor βC1 of cotton leaf curl Multan virus (CLCuMuV) for degradation [24], and CLCuMuV βC1 protein induces autophagy by disrupting the interaction of autophagy-related gene 3 (ATG3) with glyceraldehyde-3-phosphate dehydrogenases [25].

During RNA virus infection in plant, autophagy also has an antiviral function. For example, infection of turnip mosaic virus (TuMV) in *Arabidopsis thaliana* and *Nicotiana benthamiana* plants can be inhibited by the NBR1-dependent selective autophagy [26]. In addition, the core autophagic protein Beclin1 has been shown to interact with TuMV nuclear inclusion body b protein (NIb), inhibit its polymerase activity, and recruit this viral protein into autophagosomes for degradation through an interaction with ATG8a [27]. Concomitantly, plant viruses can also manipulate autophagy to fend off plant defense. For example, barley stripe mosaic virus (BSMV) γb protein can impair ATG7-ATG8 interaction to disturb autophagy-mediated antiviral defense [28]. Therefore, we consider that the arm race between virus and plant autophagy can dramatically affect plant defense against virus infection. To date, no study on the interaction between PMMoV and its host autophagy has been published.

In the present study, we investigated the global changes of gene expressions during PMMoV infection in pepper using an RNA-seq technology, and demonstrated an antiviral role of autophagy in plant responses to PMMoV infection. Our results provide new insights into the global view of RNA transcriptions after PMMoV infection in pepper and the anti-PMMoV role of host autophagy.

## Results

### Disease symptoms in PMMoV-infected pepper plants

**Fig. 1:**
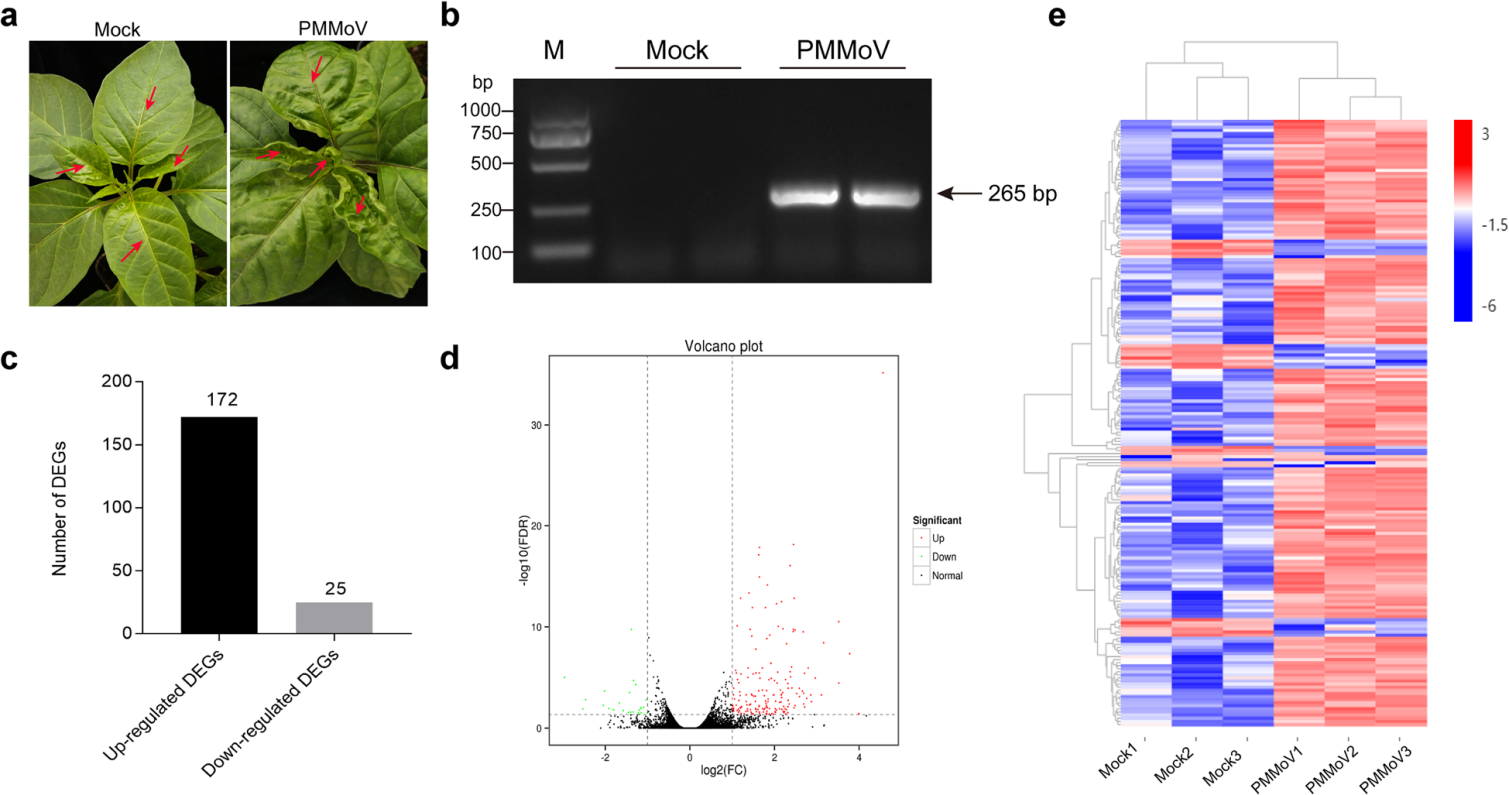
Symptoms caused by PMMoV infection in a pepper plant and transcriptomic analysis using mRNA isolated from PMMoV-inoculated and Mock-inoculated pepper plants. **a** Typical symptoms on the PMMoV-infected pepper plant. The plants were photographed at 9 days post virus or PB (Mock) inoculation. Leaves marked with red arrows were collected and used for RNA-seq. **b** PMMoV infection in the PMMoV- or Mock-inoculated plants was determined by RT-PCR using PMMoV CP gene specific primers. Lane M, *Trans*2K DNA ladder (TransGen Biotech, Beijing, China). The original data can be viewed from Additional file 11: Figure S4a. **c** A volcano plot showing differentially expressed genes (DEGs) in the PMMoV- vs Mock-inoculated pepper plants. The red and green colors represent the significantly up- and down-regulated genes, respectively (FDR < 0.05 and |log_2_ (fold change) | ≥1). **d** Total numbers of up-regulated (black) and down-regulated (gray) gene identified in this study. **e** Hierarchical clustering of DEGs based on the log_10_ FPKM values. The color scale (blue to red) represents gene expression intensities (log_10_ FPKM, low to high)

Pepper seedlings were inoculated with PMMoV-HLD isolate. Compared with the Mock-inoculated control plants, the PMMoV-inoculated pepper plants showed foliar chlorosis and distortion by 9 days post inoculation (dpi) (Fig. 1a). Infection of PMMoV in the symptomatic plants was confirmed by reverse transcription-polymerase chain reaction (RT-PCR) using PMMoV CP gene specific primers (Fig. 1b).

### RNA-seq analysis

To explore the global gene expression changes in pepper plants upon PMMoV infection, we constructed six libraries from three PMMoV-infected and three Mock-inoculated control pepper leaf samples, and analyzed them through RNA-seq. A total of 81.38 GB raw reads were obtained from these libraries (i.e., 89,803,414; 92,455,590 and 86,583,222 clean reads from three PMMoV-infected libraries, and 93,118,712; 95,261,864 and 87,522,578 clean reads from three Mock-inoculated libraries, Table 1). Among these reads, the percentage of reads mapped to the pepper genome was over 82.33% (PMMoV-infected libraries) and 83.28% (Mock-inoculated libraries), respectively (Table 1). For each library, more than 94.20% of the clean reads had a mass score at Q30 (0.1% error probability of basic calls). Furthermore, the GC content of the obtained reads ranged from 43.57 to 44.07% (Table 1).

**Table 1:** Summary of RNA-seq data

Reads	Sample					
	Mock-1	Mock-2	Mock-3	PMMoV-1	PMMoV-2	PMMoV-3
Clean Reads	93,118,712	95,261,864	87,522,578	89,803,414	92,455,590	86,583,222
Mapped Reads	77,549,891	79,662,354	73,466,998	75,027,543	76,114,634	71,325,528
	83.28%	83.62%	83.94%	83.55%	82.33%	82.38%
Unique Mapped Reads	71,992,503	74,000,161	67,056,890	69,971,154	69,015,728	66,366,868
	77.31%	77.68%	76.62%	77.92%	74.65%	76.65%
Multiple Map Reads	5,557,388	5,662,193	6,410,108	5,056,389	7,098,906	4,958,660
	5.97%	5.94%	7.32%	5.63%	7.68%	5.73%
GC Contents	43.85%	43.59%	43.90%	43.57%	44.07%	43.67%
Q30	95.08%	94.93%	94.84%	94.75%	94.60%	94.20%

### Identification of Differentially Expressed Genes (DEGs) in the PMMoV-infected pepper plants

To identify genes that play important roles during PMMoV infection in pepper plants, we analyzed the transcriptomic profiles of these six libraries and presented the expression level of each gene as FPKM, (Additional file 1). A total of 197 DEGs were identified in the PMMoV-infected libraries using the significance criteria of FDR < 0.05 and |log_2_ (FC)| ≥ 1. These DEGs included 172 up-regulated and 25 down-regulated genes (Fig. 1c, d and Additional file 1). An overview of the transcriptomic profiles and hierarchical clustering is illustrated (Fig. 1e).

### Functional enrichment analysis of DEGs

**Fig. 2:**
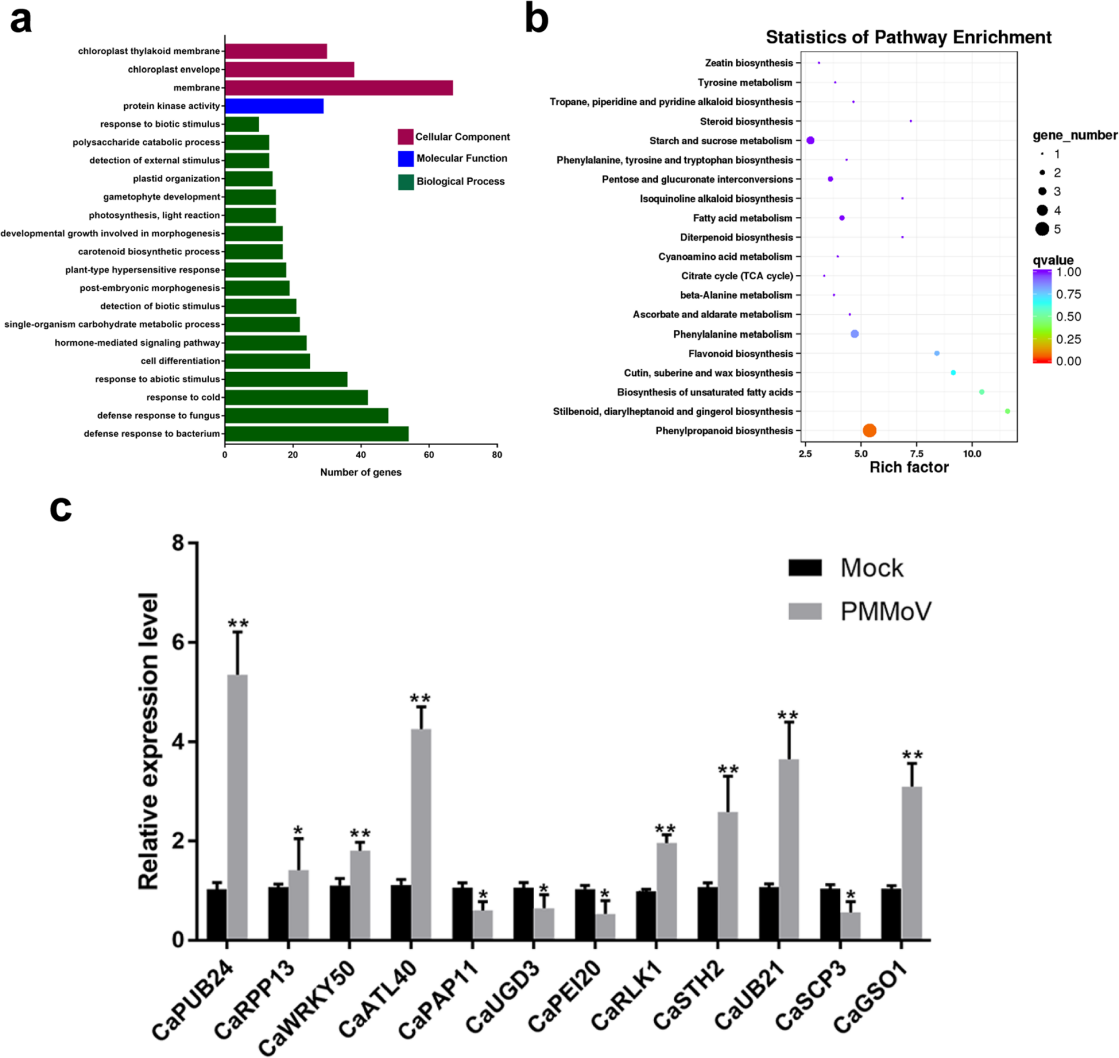
Gene Ontology (GO) and Kyoto Encyclopedia of Genes and Genomes (KEGG) analyses of DEGs responsive to PMMoV infection in pepper plants and validation of RNA-seq result. **a** GO analysis showing the GO terms in the categories of Cellular Component, Molecular Function and Biological Process (FDR < 0.05). **b** KEGG pathway enrichment analysis. The rich factor reflects the degree of enriched DGEs in given pathways. The number of enriched DGEs in various pathways is indicated by the size of the solid circle and the circle color represents the corrected *q*-value. **c** Twelve differentially expressed genes (DEGs) were selected based on the RNA-seq result and analyzed for their expressions in pepper after PMMoV infection through RT-qPCR using gene specific primers. The resulting RT-qPCR data were normalized using the expression level of *C. annuum Ubiquitin-conjugating protein* gene (*CaUBI-3*) and are presented as the means of fold change ± SD, relative to the Mock-inoculated plants. Asterisks indicate the statistically significant differences between the PMMoV- and Mock-inoculated pepper plants. (*p < 0.05, **p < 0.01)

To elucidate the main functions of DEGs, Gene Ontology (GO) analysis was performed (Additional file 2). The most significant GO terms were then used to cluster the functions of DEGs, and the GO terms (FDR < 0.05) in the three main categories were presented (Fig. 2a). Based on this result, most of the enriched GO terms in the biological process category were involved in responses to abiotic and biotic stresses (Fig. 2a and Additional file 2). In the cellular component category, the membrane and chloroplast associated GO terms were the most dominant terms. In the molecular function category, the GO term with protein kinase activity was enriched (Fig. 2a and Additional file 2).

To further dissect the molecular and biological functions of DEGs, the Kyoto Encyclopedia of Genes and Genomes (KEGG) database was used. Our results showed that the DEGs were clustered in 61 KEGG pathways (Additional file 3). Among them, six pathways were significantly enriched, with *p*-value < 0.05 (Fig. 2b and Additional file 4). The significantly enriched DEGs are those involved in the ‘stilbenoid, diarylheptanoid and gingerol biosynthesis’ (ko00945); ‘phenylpropanoid biosynthesis’ (ko00940); ‘cutin, suberine and wax biosynthesis’ (ko00073); ‘flavonoid biosynthesis’ (ko000941); ‘phenylalanine metabolism’ (ko00360); and ‘biosynthesis of unsaturated fatty acids’ (ko01040). Through the enrichment analysis of KEGG pathways, most DEGs were found to be related to the secondary metabolite biosynthesis and amino acid metabolism. This finding further indicated that PMMoV infection in pepper plants could significantly activate or deactivate the expressions of genes involved in various secondary metabolism pathways. We speculate that the secondary metabolites produced through these pathways might act to enhance plant responses to various stress conditions (Additional file 4).

### Validation of RNA-seq data

To validate the RNA-seq results, 12 DEGs related to plant defense responses were selected and tested for their expressions through RT-qPCR. These analyzed genes were: *E3 ubiquitin-protein ligase PUB24* (*CaPUB24*), *disease resistance protein RPP13* (*CaRPP13*), *WRKY transcription factor 50* (*CaWRKY50*), *RING-H2 finger protein ATL40* (*CaATL40*), *plastid-lipid-associated protein 11* (*CaPAP11*), *UDP-glucose 6-dehydrogenase 3* (*CaUGD13*), *pectinesterase inhibitor 20* (*CaPEI20*), *G-type lectin S-receptor-like serine/threonine-protein kinase RLK1* (*CaRLK1*), *pathogenesis-related protein STH-2* (*CaSTH2*), *U-box domain-containing protein 21* (*CaUB21*), *serine carboxypeptidase II-3* (*CaSCP3*) and *LRR receptor-like serine/threonine-protein kinase GSO1* (*CaGSO1*). The results showed that the expressions of *CaPUB24*, *CaRPP13*, *CaWRKY50*, *CaATL40*, *CaRLK1*, *CaSTH2*, *CaUB21* and *CaGSO1* were indeed induced upon PMMoV infection, while the expressions of *CaPAP11*, *CaUGD3*, *CaPEI20* and *CaSCP3* were down-regulated, consistent with the transcriptomic data (Fig. 2c and Additional file 1).

### PMMoV infection can induce autophagy in *N. benthamiana* plants

**Fig. 3:**
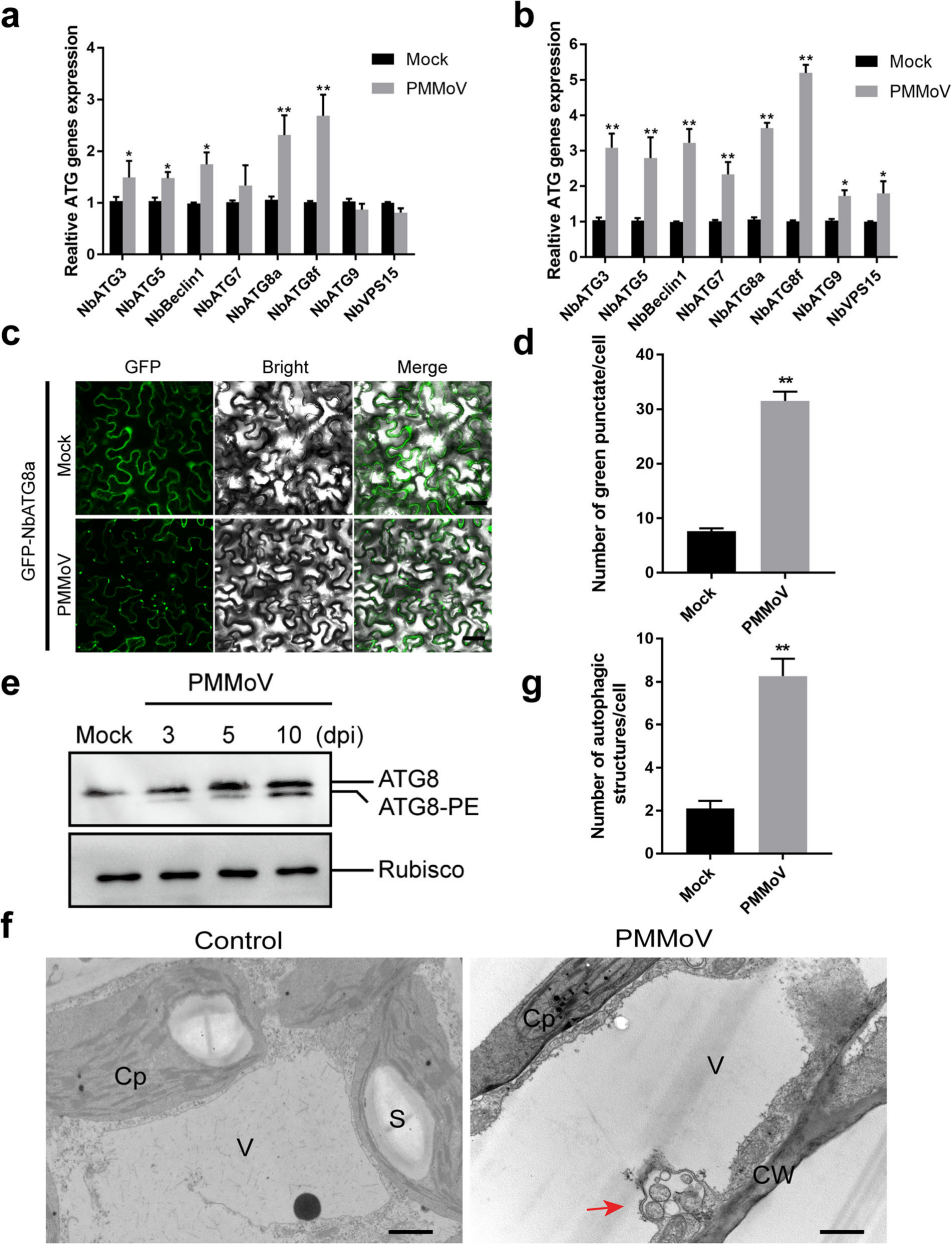
PMMoV infection activates autophagy. Quantitative RT-PCR analyses of *NbVPS15*, *NbATG3*, *NbATG5*, *NbBeclin1*, *NbATG7*, *NbATG8a*, *NbATG8f*, and *NbATG9* RNA transcript levels in the inoculated leaves at 5 dpi (**a**) and upper systemic leaves at 10 dpi (**b**) of Mock- and PMMoV-inoculated *N. benthamiana* plants, respectively. The expression level of NbActin was used as the internal control. **c** Confocal micrographs showing leaves of the PMMoV- and Mock-inoculated *N. benthamiana* plants. The leaves were infiltrated with an *Agrobacterium* culture harboring the pGD-GFP-NbATG8a plasmid and imaged at 48 h post infiltration. Numerous green fluorescence punctate were found in cells in a PMMoV-infected leaf. Bars = 30 μm. **d** Average number of green fluorescence GFP-NbATG8a punctate per 10 cells. This experiment was repeated three times and a total of 30 cells were counted for the punctate. The values represented are the mean punctate ± SD per 10 cells. **e** Total protein was isolated from young non-inoculated leaves harvested from the plants inoculated with PMMoV or PB at 3, 5 and 10 dpi, and then analyzed through Western blot using an anti-ATG8 or an anti-Rubisco antibody. The original data can be viewed from Additional file 11: Figure S4b-c. **f** Transmission electron microscopic analysis using leaf sections prepared from PMMoV- and PB (Mock)-inoculated *N. benthamiana* plants after 100 μM E64d treatment at 5 dpi. Autophagic structures (red arrows) were observed in a PMMoV-infected cell. Cp, chloroplast; CW, cell wall; S, starch; V, vacuole. Bars = 2 μm. **g** Average number of double-membrane autophagosomes ± SD in 15 cells per each treatment. The experiment was repeated twice

The above RNA-seq results have also indicated that the accumulations of several *ATG*s were up-regulated upon PMMoV infection (Additional file 5). This finding encouraged us to investigate whether autophagy was involved in defense responses to PMMoV infection. In this study, we selected PMMoV and *N. benthamiana*, a systemic host of PMMoV, as our test model. Our initial virus inoculation and RT-qPCR assays showed that the expressions of *NbATG3*, *NbATG5*, *NbBeclin1*, *NbATG8a* and *NbATG8f* in the PMMoV-inoculated *N. benthamiana* leaves were significantly up-regulated at 5 dpi (Fig. 3a). Similar results were also found in the systemic leaves of the PMMoV-inoculated *N. benthamiana* plants at 10 dpi (Fig. 3b). We then analyzed the effect of PMMoV infection on autophagy through transient expression of a GFP:NbATG8a fusion protein in the PMMoV-infected or non-infected *N. benthamiana* leaves. Compared with the non-infected *N. benthamiana* leaves, the number of green fluorescent punctate, resembling the pre-autophagosomal or autophagosomal structures, was increased by more than two-fold in the PMMoV-infected *N. benthamiana* leaves (Fig. 3c, d). Consistently, high levels of ATG8 proteins were detected in PMMoV-infected *N. benthamiana* plants by immunoblotting. The conversion of ATG8 to ATG8-PE is generally considered to be a reliable indicator of autophagy. At 5 and 10 dpi, the accumulation level of ATG8-PE was increased notably in PMMoV-infected *N. benthamiana* plants by immunoblot assays (Fig. 3e). Transmission electron microscopy (TEM) results showed that PMMoV infection in *N. benthamiana* plants induced numerous autophagic structures in leaf cells, compared with the non-infected *N. benthamiana* leaf cells (Fig. 3f, g). Taken together, these results demonstrated that PMMoV infection can induce autophagy in *N. benthamiana* plants.

### Inhibition of autophagy can increase PMMoV RNA accumulation in *N. benthamiana*

**Fig. 4:**
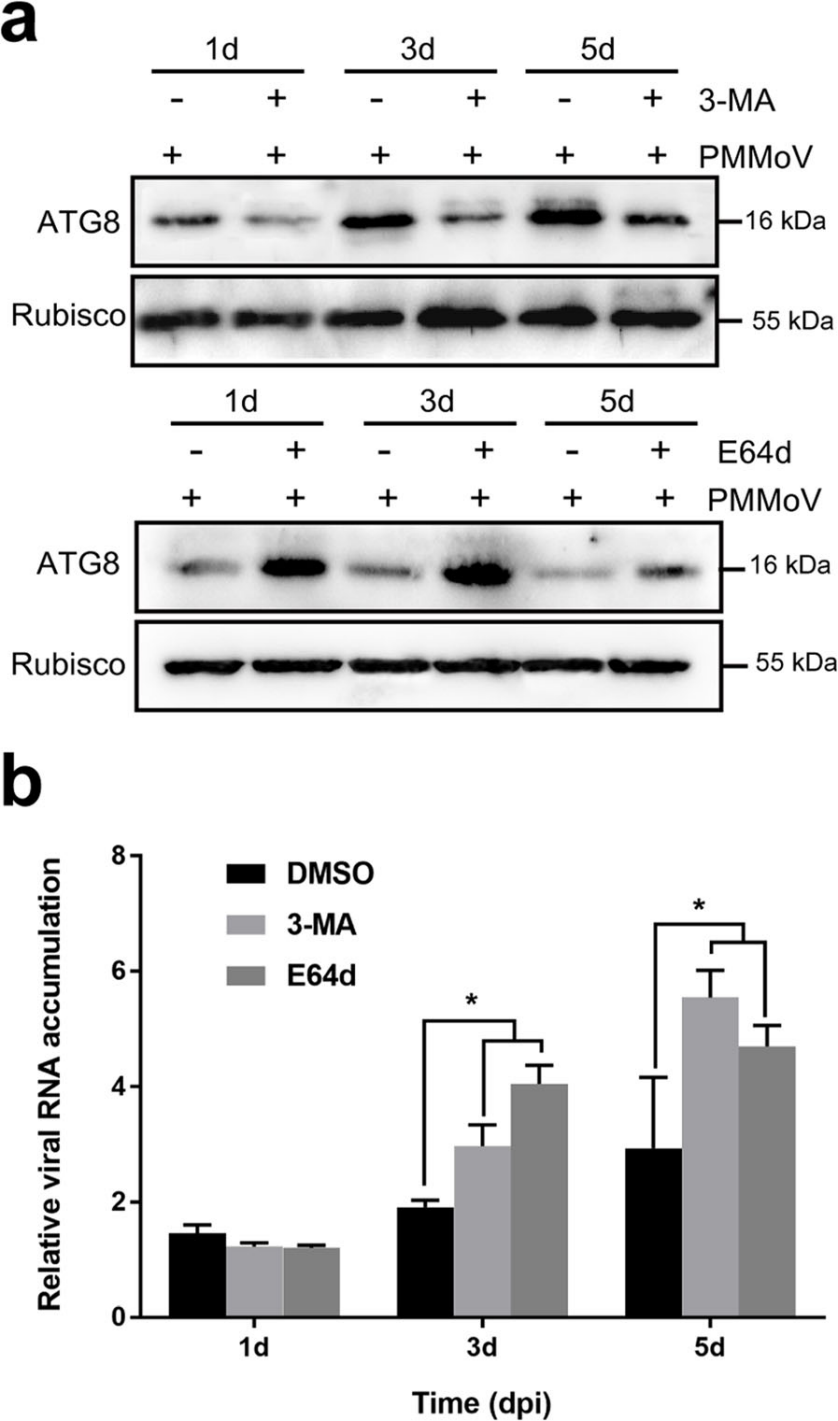
Effects of 3-MA and E64d on PMMoV RNA accumulation. **a** Total protein was isolated from PMMoV-infected *N. benthamiana* leaves treated with 3-MA, E64d or DMSO (mock). The protein samples were analyzed through Western blot assays using an anti-ATG8 or an anti-Rubisco antibody. The original data can be viewed from Additional file 11: Figure S4d-g. **b** Effect of 3-MA and E64d treatment on PMMoV infection. PMMoV-infected leaves treated with 3-MA, E64d or DMSO (mock) were harvested at 1 dpi, 3 dpi and 5 dpi, respectively. Total RNA was isolated from these leaf samples and analyzed through RT-qPCR. Values presented are the means of fold change ± SD, relative to that in the DMSO-treated control plants. Asterisks indicate statistical difference between treatments, determined by the Student’s *t*-test (*p < 0.05)

To investigate the role of autophagy in PMMoV infection, *N. benthamiana* plants were first inoculated with PMMoV or with phosphate buffer only (Mock). Before sampling, the second leaf above the inoculated leaves of each assayed plant was treated with an autophagy inhibitor. At 1, 3 or 5 dpi, the inhibitor-treated leaves were harvested and then analyzed for NbATG8 accumulation through Western blot assay. Leaves from the plants treated with DMSO only were used as the controls. Our results showed that after 3-MA treatment, the accumulation level of NbATG8 was significantly decreased (Fig. 4a). In contrast, the treatment with E64d significantly increased the level of NbATG8, compared with that in the DMSO-treated plant leaves (Fig. 4a). We then analyzed PMMoV RNA accumulation in the 3-MA-, E64d- or DMSO-treated plant leaves through RT-qPCR. Our results showed that the PMMoV RNA level was significantly increased in the 3-MA- or E64d-treated plants at 3 and 5 dpi (Fig. 4b), indicating that inhibition of autophagy can increase plant susceptibility to PMMoV infection.

### Knockdown of *NbATG3*, *NbATG5*, *NbBeclin1*, *NbATG7* or *NbATG8a* expression promotes PMMoV infection in *N. benthamiana*

**Fig. 5:**
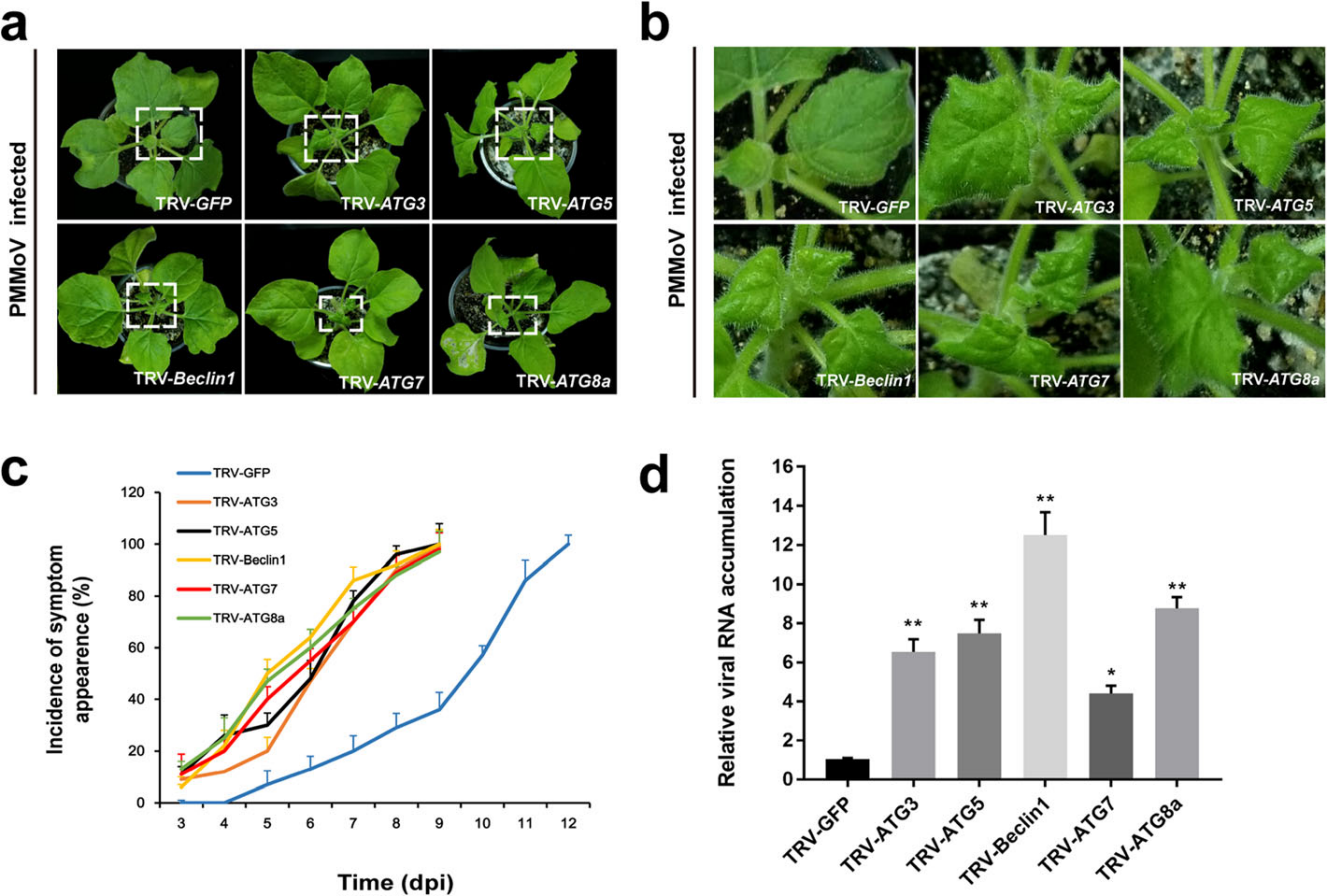
Silencing of five key *ATG*s expressions in *N. benthamiana* plants through VIGS vectors enhances PMMoV infection. **a** Disease symptoms on the PMMoV-inoculated, *NbATG3*-, *NbATG5*-, *NbBeclin1*-, *NbATG7*- or *NbATG8a*-silenced *N. benthamiana* plants. The PMMoV-inoculated TRV-GFP-infiltrated *N. benthamiana* plants were used as controls. **b** Close-up views of upper leaves indicated by white dash boxes in (**a**). **c** Percentages of PMMoV infection in various *ATG*-silenced plants or in the TRV-GFP-inoculated plants at various days post PMMoV inoculation. Values presented are the means ± SD from three independent experiments. **d** Relative accumulation of PMMoV RNA in various silenced and non-silenced plants at 7 days post PMMoV inoculation. Values presented are the means ± SD from three biological replicates. Asterisks indicate statistical difference between treatments, determined by the Student’s *t*-test (*p < 0.05, **p < 0.01)

Autophagy is known to play an antiviral role in plant [29]. To further investigate the effect of autophagy on PMMoV infection, we silenced *NbATG3*, *NbATG5*, *NbBeclin1*, *NbATG7* and *NbATG8a* expressions in *N. benthamiana* plants individually using specific TRV-based VIGS vectors. Plants inoculated with the TRV vector carrying a 205 bp GFP gene insert were used as controls. At 10 days post agroinfiltration (dpai), the third and fourth leaves above the agroinfiltrated leaves on each assayed plant were rub-inoculated with PMMoV-infected crude leaf extracts. After 7 days post PMMoV inoculation, the *NbATG3*-, *NbATG5*-, *NbBeclin1*-, *NbATG7*- or *NbATG8a*-silenced plants showed much stronger PMMoV symptoms on their systemic leaves than the non-silenced control plants (Fig. 5a, b). In addition, the systemic PMMoV symptoms on the gene-silenced plants appeared quicker than that on the non-silenced plants (Fig. 5c). Silencing efficiency of each target gene and PMMoV RNA accumulation were determined through RT-qPCR using the first and second systemic leaf tissues harvested from each assayed plant at 7 dpi with PMMoV, respectively (Fig. 5d and Additional file 6). The results showed that knockdown of these *ATG* gene expressions individually enhanced PMMoV accumulation, especially in the *NbBeclin1*-silenced plants (Fig. 5d), further supporting the notion that autophagy play an important anti-PMMoV infection role in plants.

## Discussion

PMMoV infection is a great threat to pepper industry. Therefore, development of effective control strategies is crucial to pepper farmers. It is well known that successful virus infection in plant depends on the outcome of arm race between virus and host plant, and autophagy plays crucial roles in the arm race [30–32]. To date, how PMMoV modulates host autophagy is unclear. Since the first report of transcriptome sequencing, RNA-seq technology has been heavily and successfully used to explore host genes involved in the interactions between viruses and host plants [33–35].

Through transcriptome sequencing, we have obtained a detailed information on the changes of global gene expressions in pepper plants after PMMoV infection. Through comprehensive analysis of these RNA-seq data, we have identified 197 DEGs (Fig. 1c), associated with different regulatory pathways. We speculate that some or many DEGs, associated with host defense responses, play important roles in pepper responding to PMMoV infection. For example, through GO and KEGG pathway analyses, multiple stress responsive genes or genes associated with plant-pathogen interaction pathways were induced upon PMMoV infection (Fig. 2). The DEG induction cascades may lead to incompatible interactions between pepper plant and PMMoV (Additional file 1), resulting in mild PMMoV symptoms (Fig. 1a). These identified DEGs are mostly associated with kinase signal transductions, transcription factors (TFs) and defense response genes. Activation of host defense responsive genes can lead to the production of antimicrobial secondary metabolites that can inhibit further spread of PMMoV in pepper plants.

Virus-induced pathogen associated molecular pattern (PAMP)-triggered immunity plays important roles in plant immunity to virus infection [32]. Plant PUB24 can act, in concert with PUB22 and PUB23, as a negative regulator of PTI in the response to several PAMPs [36]. Because the expression of *CaPUB24* was induced after PMMoV infection in pepper (Fig. 2 and Additional file 1), we hypothesize that the induction of *CaPUB24* expression may decrease PTI response and defense responses. Receptor-like protein kinases (RLKs) and receptor-like proteins (RLPs) are members of the serine/threonine protein kinase family, and are known to be involved in sensing external signals [37, 38]. In this study, the expression level of *CaRLK1* was significantly up-regulated after PMMoV infection (Fig. 2 and Additional file 1). As a receptor of pathogen signaling, leucine-rich repeat (LRR) receptor-like serine/threonine-protein kinase GSO1 was also up-regulated after PMMoV infection (Fig. 2 and Additional file 1). Pathogenesis-related (PR) proteins are well-known plant defense proteins against both biotic and/or abiotic stresses, especially pathogen infections [39]. In this work, we have found that the expressions of *PR* genes: a tobacco mosaic virus (TMV) resistance protein gene and three disease resistance protein genes, were altered after PMMoV infection in pepper (Additional file 1). Although the expression level of *CaSTH2* was induced upon PMMoV infection (Fig. 2 and Additional file 1), an earlier study showed that over-expression of STH2 in transgenic potato plants did not affect potato virus X infection [40], suggesting that *CaSTH2* may not play a direct role in pepper plant against PMMoV infection. Plant disease resistance proteins can indirectly interact with specific avirulence proteins produced by pathogens. In contrast to other resistance proteins, RPP13 does not require salicylic acid accumulation, and act independently of ESD1 and NSD1 that are two independent signaling pathways that confer gene-for-gene resistance in *Arabidopsis* [41, 42]. In this study, the expression of *CaRPP13* was up-regulated after PMMoV infection, suggesting the existence of an independent signaling pathway in response to PMMoV infection (Fig. 2 and Additional file 1). Many TFs have been shown to be involved in plant defense signaling pathways [43]. In this study, the expressions of seven *CaWRKY* genes were found to be induced after PMMoV infection (Fig. 2 and Additional file 1). Previous studies have shown that *WRKY3*, *WRKY70* and *WRKY75* are involved in plant-pathogen interactions [44, 45]. Therefore, we speculate that these seven *WRKY* genes may also play important roles in early pepper response to PMMoV infection.

**Fig. 6:**
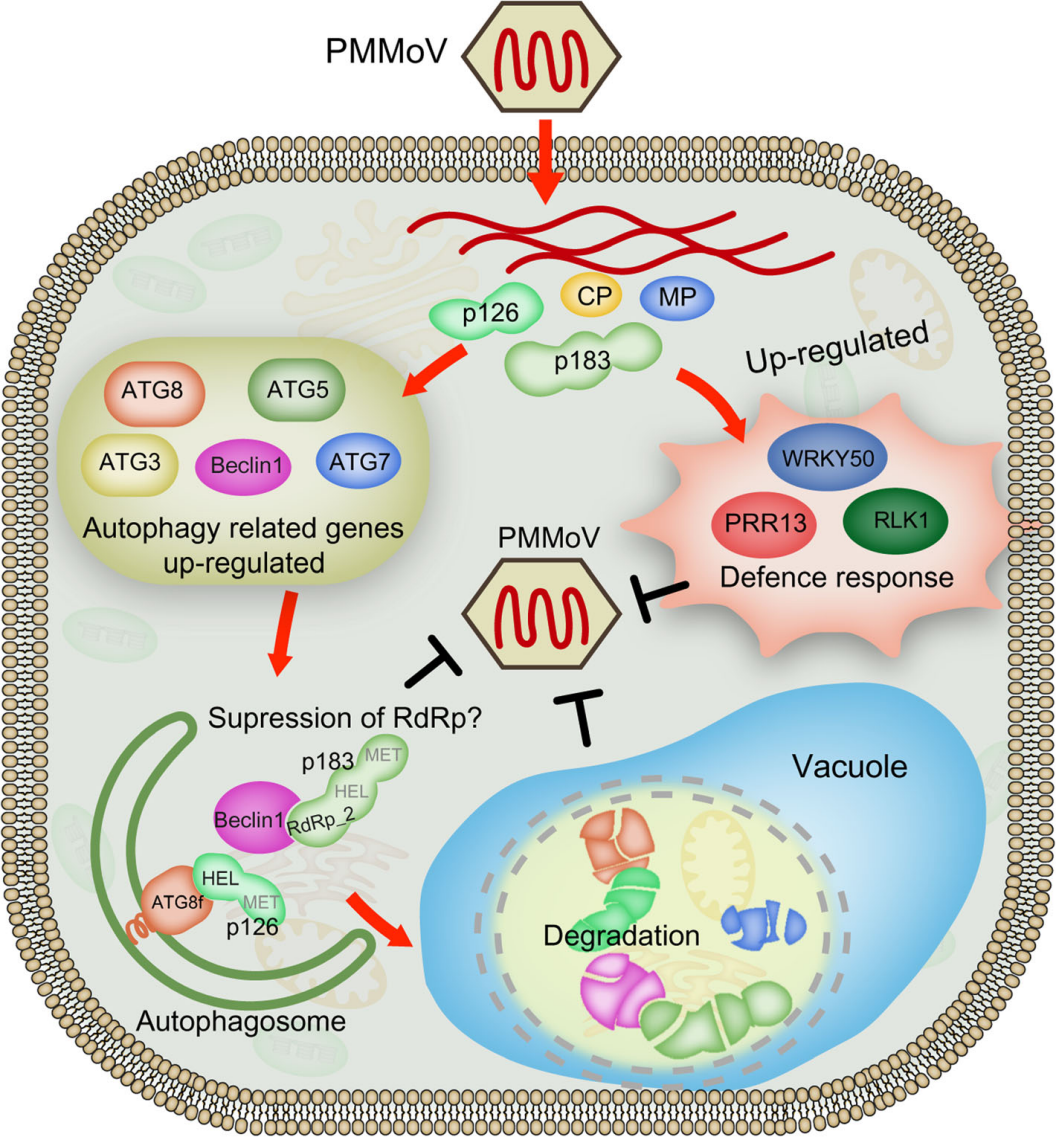
Proposed model for the possible roles of autophagy and gene regulation involved in resistance against PMMoV infection

Several recent studies on plant autophagy and pathogen infections have revealed the importance of autophagy during pathogen infections [29, 46]. Other studies have shown the specific roles of autophagy in eliminating viruses in mammalian cells [47, 48]. On the other hand, several plant viruses have been shown to inhibit or utilize autophagy to promote their infections in plants [26, 28, 49–51]. Here, we show that autophagy plays an important role in pepper defense responses to PMMoV infection. Our conclusion is based on the fact that by 9 days post PMMoV inoculation, the expressions of 17 autophagy-associated genes were altered in the pepper plants, although the expression changes of several genes were not statistically significant (Additional file 5). It is noteworthy that the RT-qPCR analysis done at 14 dpi showed that six out of the eight analyzed autophagy-associated genes were significantly up-regulated and one was significantly down-regulated (Additional file 7), suggesting strongly that autophagy plays a role in pepper defense responses to PMMoV infection. In mammalian cells, defective ATG5 or ATG7 is unable to prevent autolysosome formations, which are known to be induced by various stresses. It is recommended that macroautophagy can occur through at least two pathways: a conventional ATG5/ATG7-dependent pathway and an atypical ATG5/ATG7-independent pathway [52]. Our results suggest that the autophagy induced by PMMoV infection is likely a conventional ATG5/ATG7-dependent pathway. Several reports have indicated the involvement of ATG8 family proteins in autophagy biogenesis and cargo recruitment [53]. In this study, we further determined that silencing of *NbATG3*, *NbATG5*, *NbBeclin1*, *NbATG7* or *NbATG8a* expression in *N. benthamiana* plants through VIGS vectors can enhance PMMoV RNA accumulation and disease symptom formation (Fig. 5). To explore the role of autophagy in PMMoV infection and provide a basis for further study on the relationship between autophagy and PMMoV, we analyzed the physiological significance of autophagy on PMMoV viral accumulation. By inhibiting the autophagy pathway with treatment of 3-MA or E64d, we demonstrated that PMMoV infection activated the autophagy, which was utilized by plants to inhibit PMMoV infection (Fig. 4). However, these two inhibitors had opposite effects on the accumulation of ATG8 protein (Fig. 5). 3-MA can inhibit the formation of autophagosomes at the initial stage of autophagy [54], while E64d can stabilize the autophagosomes in the vacuoles to prevent its degradation at the later stage of autophagy [55], which results in a contradictive accumulation of ATG8 protein. In conclusion, we propose that autophagy plays an important role in plant defense responses to PMMoV infection (Fig. 6).

As a core component of the class III PI3K complex, Beclin1 can regulate autophagy through acting as the interaction center for different proteins [56]. Moreover, Beclin1/ATG6 complex is required during autophagy in plant, human and other eukaryotes [57]. A previous study by Liu and others has shown that silencing the expressions of *Beclin1* and several other *ATG* genes in *N. benthamiana* plants through VIGS vectors enhanced TMV infection [22]. The Beclin1-mediated autophagy may also inhibit TMV infection through an interaction with TMV encoded RNA-dependent RNA polymerase (RdRp). Viral RdRp is necessary for RNA virus replication, and all the known viral RdRps have conserved GDD motifs [58]. To further explore defense responses triggered by autophagy to PMMoV infection, we investigated the interaction between the conserved GDD motif in PMMoV RdRp_2 and NbBeclin1 through a yeast two-hybrid technique. As expected, PMMoV RdRp_2 did interact with NbBeclin1 (Additional file 8), suggesting that NbBeclin1 could directly bind with PMMoV RdRp_2 and suppress RdRp activity to restrict viral infection. Particularly, our findings further demonstrated that targeting viral RdRp by Beclin1 is a conserved antiviral mechanism in plants (Fig. 6). Meanwhile, our results showed that the HEL motif in PMMoV p126 could interact with NbATG8f (Additional file 8), suggesting that p126 may be a novel target for autophagic degradation by binding to NbATG8f (Fig. 6), which need to be further investigated.

## Conclusions

In summary, we have analyzed pepper responses to PMMoV infection through an RNA-seq approach. Our results indicate that several genes responsible for signal transductions or stress responses may play important roles in defense responses to PMMoV infection (Fig. 6). In addition, we have shown that the expressions of many *ATGs* in pepper plants were up-regulated after PMMoV infection. Further studies have shown that PMMoV infection can induce autophagy in cells, and autophagy plays an antiviral role during the arm race between host plant and PMMoV (Fig. 6). These results presented in this paper should help us to better understand the mechanism controlling PMMoV infection in plant and to develop better strategies for breeding projects for virus-resistant crops.

## Methods

### Plasmid construction

Sequences of seven *N. benthamiana* genes: *NbBeclin1* (accession number AY701316), *NbVPS15* (KU561371), *NbATG9* (KX369399), *NbATG3* (KX369396), *NbATG5* (KX369397), *NbATG7* (KX369398) and *NbATG8a* (KX120976), were retrieved from the GenBank database (https://www.ncbi.nlm.nih.gov/genbank/). Full-length *NbATG8a* gene was PCR amplified and cloned behind an *eGFP* gene in a pGD vector to express a GFP:NbATG8a fusion protein. Sequences of primers used in plasmid construction are shown in Additional file 9.

### Plant growth and virus inoculation

Seeds of *Capsicum annuum* L. cultivar ‘Zunla-1’ were kindly provided by Dr. Dan Xing (Guizhou Academy of Agricultural Sciences, Guiyang, China). Wild-type *N. benthamiana* seeds were propagated and stored in the Plant Virus Laboratory, Shenyang Agricultural University, Shenyang, China. All the seeds were grown inside a growth chamber set at 25 °C and a 16 and 8 h (light/dark) photoperiod and 60% humidity. PMMoV-HLD isolate was from a previously reported source [59] in our lab. For virus inoculation, approximately 0.5 g of PMMoV-infected leaf tissues were homogenized in phosphate buffer (PB, 0.01 M, pH 7.2) at 1:5 (w/v) ratio. The crude leaf extract was rub-inoculated to the upper two young leaves of 4-leaf stage pepper plants. At 9 days post virus inoculation (dpi), the upper un-inoculated young leaves (Fig. 1a, red arrows) were harvested, frozen in liquid nitrogen, and then stored at − 80 °C till further use. Leaves of plants inoculated with PB only were also harvested at 9 dpi and used as the non-infected controls.

### RNA isolation and Rreverse Transcription-Polymerase Chain Reaction (RT-PCR)

To detect PMMoV infection in pepper plants, total RNA was extracted from collected leaf samples using the RNAiso Plus reagent (TaKaRa, Dalian, China) as instructed. Concentrations and qualities of the isolated RNA samples were monitored using a NanoDrop2000 spectrophotometer (Thermo Fisher Scientific, Waltham, USA). First-strand cDNAs were synthesized using 1 μg of total RNA, an M-MLV reverse transcriptase (Promega, Carlsbad, USA), and an equal volume of oligo (dT) and random primers (2.5 μM) in a final volume of 20 μL according to the manufacturer’s instructions. The PCR amplification was performed in a final volume of 25 μL containing 2.5 units of PrimeSTAR® Max DNA polymerase (TaKaRa, Dalian, China), 1 μL of each PMMoV CP gene specific primers (10 μM) (Additional file 9) and 2 μL of cDNA template. Conditions of PCR reaction was 35 cycles of 98 °C for 10 s, 55 °C for 5 s, and 72 °C for 20 s.

### RNA sequencing (RNA-seq)

For one virus inoculation experiment, the upper young leaves (Fig. 1a, red arrows) from at least 15 individual PMMoV-infected and non-infected pepper plants were pooled and used for RNA-seq, respectively. Three independent virus inoculation experiments were performed in this study. One microgram of total RNA from each sample was used as an input for RNA preparation. Sequencing libraries were constructed using the NEBNext®Ultra™ RNA Library Prep kit (New England Biolabs, Ipswich, USA) and the NEB Next Multiplex Oligos (New England Biolabs) followed by sequencing on an Illumina HiSeq 4000 sequencing platform (Biomarker Biology Technology, Beijing, China) as instructed. Briefly, mRNA was prepared using the poly-T oligo-attached magnetic beads. After fragmentation, cDNAs were synthesized and then ligated with the NEB Next Adaptors with hairpin loop structures. The resulting PCR products were purified and the quality of the libraries was assessed using the Agilent Bioanalyzer 2100 system.

The clean reads were aligned individually against the reference genome of *Capsicum annuum*. L_Zunla-1_Release_2.0 (http://peppersequence.genomics.cn) using the TopHat 2.0 software [60]. The number of clean reads mapped to a specific gene were counted and then presented as fragments per kilobase of transcript per million mapped reads (FPKM) using Cufflinks [61]. Differentially expressed genes (DEGs) between the PMMoV-infected and the non-infected control samples (Mock) were identified using the DESeq software [62]. The DEGs were ranked individually based on the average log_2_ Fold Change (FC) and the false discovery rate (FDR) Q values, and then filtered using FDR Q < 0.05 and |log_2_ FC| ≥1. Gene function was annotated according to the following databases: Gene Ontology (GO), Kyoto Encyclopedia of Genes and Genomes (KEGG) Ortholog (KO), Swiss-Prot, and NCBI non-redundant protein sequences (Nr). GO enrichment of DEGs was determined based on the Wallenius noncentral hypergeometric distribution using the GOseq R package [63].

### Reverse Transcription-Quantitative PCR (RT-qPCR)

To validate the RNA-seq results, 12 DEGs were selected and tested for their expressions through RT-qPCR. Three micrograms of total RNA (each sample) was used for cDNA synthesis using the HiScript III RT SuperMix kit (Vazyme, Nanjing, China) followed by qPCR amplification using the AceQ qPCR SYBR Green Master Mix kit (Vazyme) on the StepOne plus real time PCR platform (Applied Biosystems, Foster City, USA). Primers used in qPCR amplifications were designed according to the gene sequences from the *Capsicum annuum*. L_Zunla-1_Release_2.0 database (http:// peppersequence.genomics.cn) and RNA-seq data. The primers were synthesized by the Sangon Biotech, Shanghai, China (Additional file 10). The expression levels of *C. annuum Ubiquitin-conjugating protein* gene (*CaUBI-3*, AY486137) [64] or *N. benthamiana Actin* gene (*NbActin*, AY179605) was used as internal controls, respectively. Relative expression levels of these genes were calculated using the 2^-ΔΔCT^ method [65]. Three biological and three technical replicates were performed for all RT-qPCR experiments.

### Confocal microscopy and Transmission Electron Microscopy (TEM)

Fully expanded leaves of 3-week-old *N. benthamiana* plants were rub-inoculated with PMMoV-infected crude leaf extract. After 5 dpi, the second leaf above the inoculated leaves was infiltrated with *Agrobacterium tumefaciens* strain GV3101 (OD_600_ = 0.6) containing the recombinant pGD vector expressing GFP:NbATG8a fusion. At 48 h post agroinfiltration (hpai), the infiltrated leaf areas were sampled and examined under an Olympus Fluoview FV3000 confocal laser scanning microscope (Olympus, Tokyo, Japan). The excitation wavelength was set at 488 nm and the emission was captured at 522 nm.

At 5 dpi, a systemic leaf of the PMMoV- or Mock-inoculated *N. benthamiana* plants was treated with 100 μM E64d diluted in a DMSO solution or with DMSO solution alone (mock) for 10 h. The treated leaves were then harvested and cut into small segments (1–2 mm^2^) for electron microscopic analysis. The tissue fixation, embedding, sectioning and TEM observation were as described [66].

### Chemical treatments

Autophagy inhibitors 3-methyladenine (3-MA; Sigma, Darmstadt, Germany) and E64d (Abcam, Eugene, USA) were used to investigate the effects of autophagy on PMMoV infection. 3-MA disturbs early autophagosome formation due to the suppression of class III PtdIns 3-kinase, and E64d can stabilize autophagic bodies in the vacuole to inhibit autophagy at late stage [54, 55]. Infiltration buffer containing dimethyl sulfoxide (DMSO; Mock) or an equal volume of DMSO with 5 mM 3-MA, or 100 μM E64d for inhibition of autophagy, was infiltrated into leaves for 10 h before samples were collected.

### Western blot analysis

Total protein was extracted from agroinfiltrated leaves as described previously [67]. Concentrations of total protein in different samples were quantified using the bicinchoninic acid (BCA) method followed by electrophoresis in 10% SDS-PAGE gels. For ATG8 detection, the denatured proteins were separated on a 15% SDS-PAGE gel in the presence of 6 M urea. For western blotting, the separated protein bands were transferred onto 0.45 μm polyvinylidene fluoride (PVDF) membranes (Millipore, Billercia, USA). Then, the membranes were blocked with a 5% Difco™ skim milk (Becton Dickinson and Company, Sparks, USA) solution and then probed overnight with an antibody specific for ATG8 (Agrisera, Vännäs, Sweden) or Rubisco (Abbkine, California, USA) at 4 °C. After several rinses in the Tris-buffered saline supplemented with 0.1% Tween 20 (TBST), the membranes were incubated again in a horseradish peroxidase (HRP)-conjugated goat anti-rabbit IgG solution (ZSGB-BIO, Beijing, China) for 1 h at room temperature. After several rinses in the TBST buffer, the detection signal was visualized by incubating the membranes in a chemiluminescent detection reagent kit as instructed (Millipore).

### Virus-Induced Gene Silencing (VIGS) assay

For VIGS assays, fragments representing partial sequence of *NbBeclin1* (343 bp), *NbATG8a* (265 bp), *NbATG3* (329 bp), *NbATG5* (317 bp) or *NbATG7* (324 bp) were RT-PCR-amplified and inserted individually into a *Tobacco rattle virus* (TRV)-based VIGS vector. Briefly, these fragments were amplified individually from an *N. benthamiana* cDNA through PCR using specific primers (Additional file 10) and the PrimeSTAR® Max DNA Polymerase (TaKaRa). The resulting PCR fragments were digested with the *BamH* I and *Xho* I restriction enzymes and cloned individually into the pTRV2 plasmid to produce pTRV2-Beclin1, pTRV2-NbATG8a, pTRV2-NbATG3, pTRV2-NbATG5 and pTRV2-NbATG7, respectively. These new plasmids and the pTRV1 plasmid were transformed individually into *Agrobacterium tumefaciens* strain GV3101 cells. After propagation, each *Agrobacterium* culture was pelleted and then resuspended in an infiltration buffer containing 10 mM MES, 10 mM MgCl_2_, pH 7.2, and 150 μM Acetosyringone. *Agrobacterium* culture harboring pTRV1 was mixed with an equal amount of *Agrobacterium* culture harboring pTRV2-Beclin1, pTRV2-NbATG8a, pTRV2-NbATG3, pTRV2-NbATG5 or pTRV2-NbATG7. The mixed cultures were referred to as TRV-NbATG3, TRV-NbATG5, TRV-NbATG7, TRV-NbATG8a, and TRV2-Beclin1, respectively. The final optical density (OD_600_) of each mixed *Agrobacterium* culture was 0.5, and the cultures were infiltrated individually into leaves of 4–5 leaf stage *N. benthamiana* plants using needleless syringes. At 10 days post agroinfiltration (dpai), the upper non-infiltrated *N. benthamiana* leaves were inoculated with PMMoV-infected crude *N. benthamiana* leaf extracts or PB only (Mock).

## Supplementary Information

**Additional file 1: Table S1.** Annotation of DEGs between Mock- vs PMMoV-inoculated pepper plants.

**Additional file 2: Table S2.** Gene ontology (GO) annotation of DEGs.

**Additional file 3: Table S3.** KEGG annotation of DEGs.

**Additional file 4: Table S4.** Enriched PMMoV-related KEGG pathways.

**Additional file 5: Table S5:** The expressions of autophagy-associated genes in pepper plants upon PMMoV infection.

**Additional file 6: Figure S1.** Silencing efficiency of five *ATG*s in *N. benthamiana* plants through VIGS vectors.

**Additional file 7: Figure S2.** Relative expressions of eight *ATG*s in pepper plants infected with PMMoV.

**Additional file 8: Figure S3.** Analysis of interaction between PMMoV viral proteins and autophagy proteins through yeast two hybrid assay.

**Additional file 9: Table S6.** Primers used for PMMoV detection and vector construction.

**Additional file 10: Table S7.** Primers used for RT-qPCR in this study.

**Additional file 11: Figure S4.** Source data for Fig. 1b, Fig. 5e and Fig. 6a. (a) Red frame displayed the source data for Fig. 1b. (b-c) Full scan of results shown in Fig. 5e. (d-g) Full scan of the results shown in Fig. 6a.

## Data Availability

The raw data collected from RNA-seq was availability in national center for biotechnology information (NCBI): https://dataview.ncbi.nlm.nih.gov/objects?linked_to_id=PRJNA631504&archive=biosample. SRA accession: PRJNA631504. The datasets used and/or analysed during the current study are available from the corresponding author on reasonable request.

## Abbreviations

ATG: Autophagy-related gene
BSMV: Barley stripe mosaic virus
CaMV: Cauliflower mosaic virus
CLCuMuV: Cotton leaf curl Multan virus
DEG: Differentially expressed gene
DMSO: Dimethyl sulfoxide
Dpai: Days post agroinfiltration
FPKM: Fragments per kilobase of transcript per million mapped reads
Hpai: Hours post agroinfiltration
PCD: Programmed cell death
PMMoV: Pepper mild mottle virus
PVDF: Polyvinylidene fluoride
RdRp: RNA-dependent RNA polymerase
TEM: Transmission electron microscopy
TMV: Tobacco mosaic virus
TRV: Tobacco rattle virus
TuMV: Turnip mosaic virus
