# Supplementary figures and images for: Transcriptomic and functional analyses reveal an antiviral role of autophagy during pepper mild mottle virus infection
Source: BMC Plant Biol. 2020 Oct 29;20:495. doi: 10.1186/s12870-020-02711-x (PMC7596970; doi:10.1186/s12870-020-02711-x)

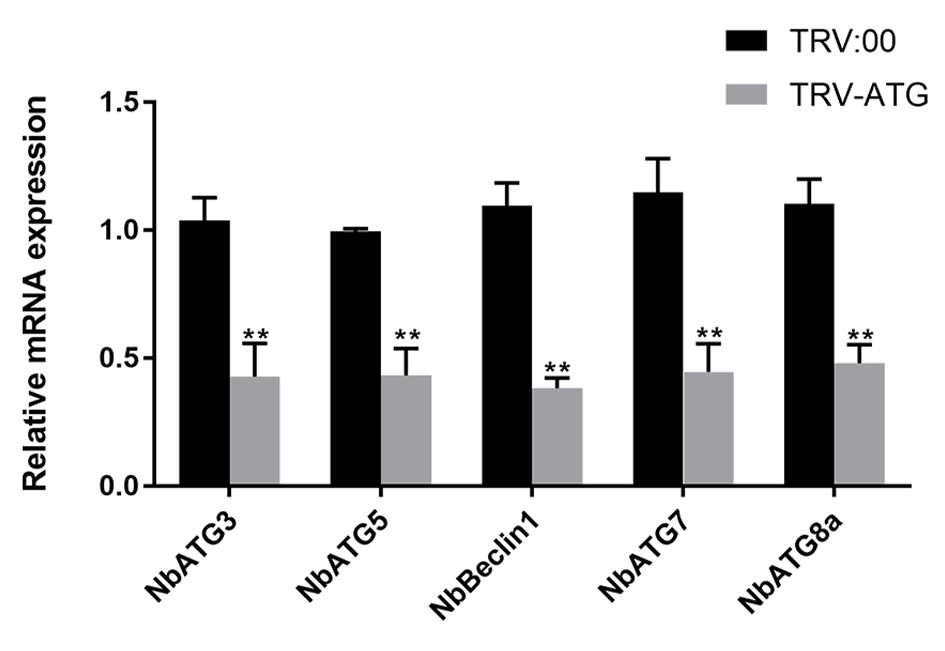

Supplement: Supplementary file 6 — Additional file 6: Figure S1. Silencing efficiency of five ATGs in N. benthamiana plants through VIGS vectors. [file 12870_2020_2711_MOESM6_ESM.tif]

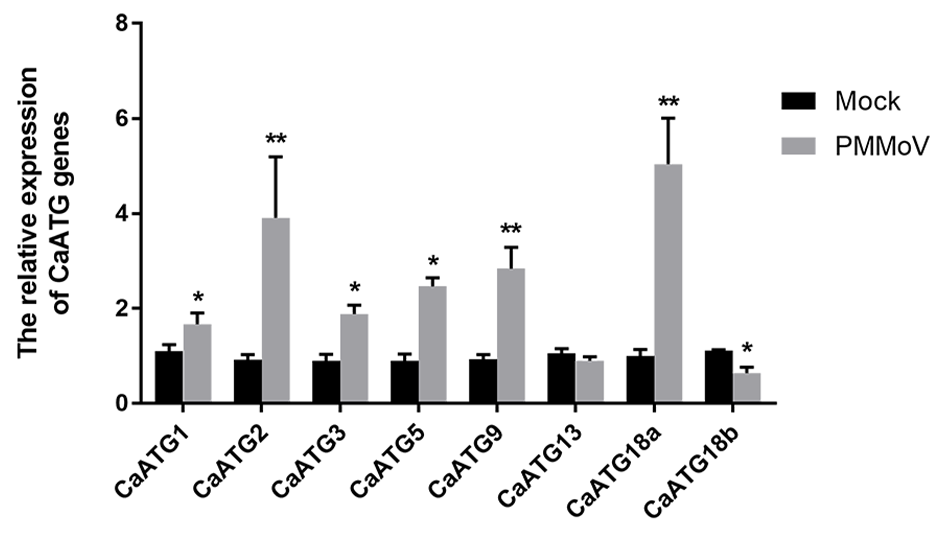

Supplement: Supplementary file 7 — Additional file 7: Figure S2. Relative expressions of eight ATGs in pepper plants infected with PMMoV. [file 12870_2020_2711_MOESM7_ESM.tif]

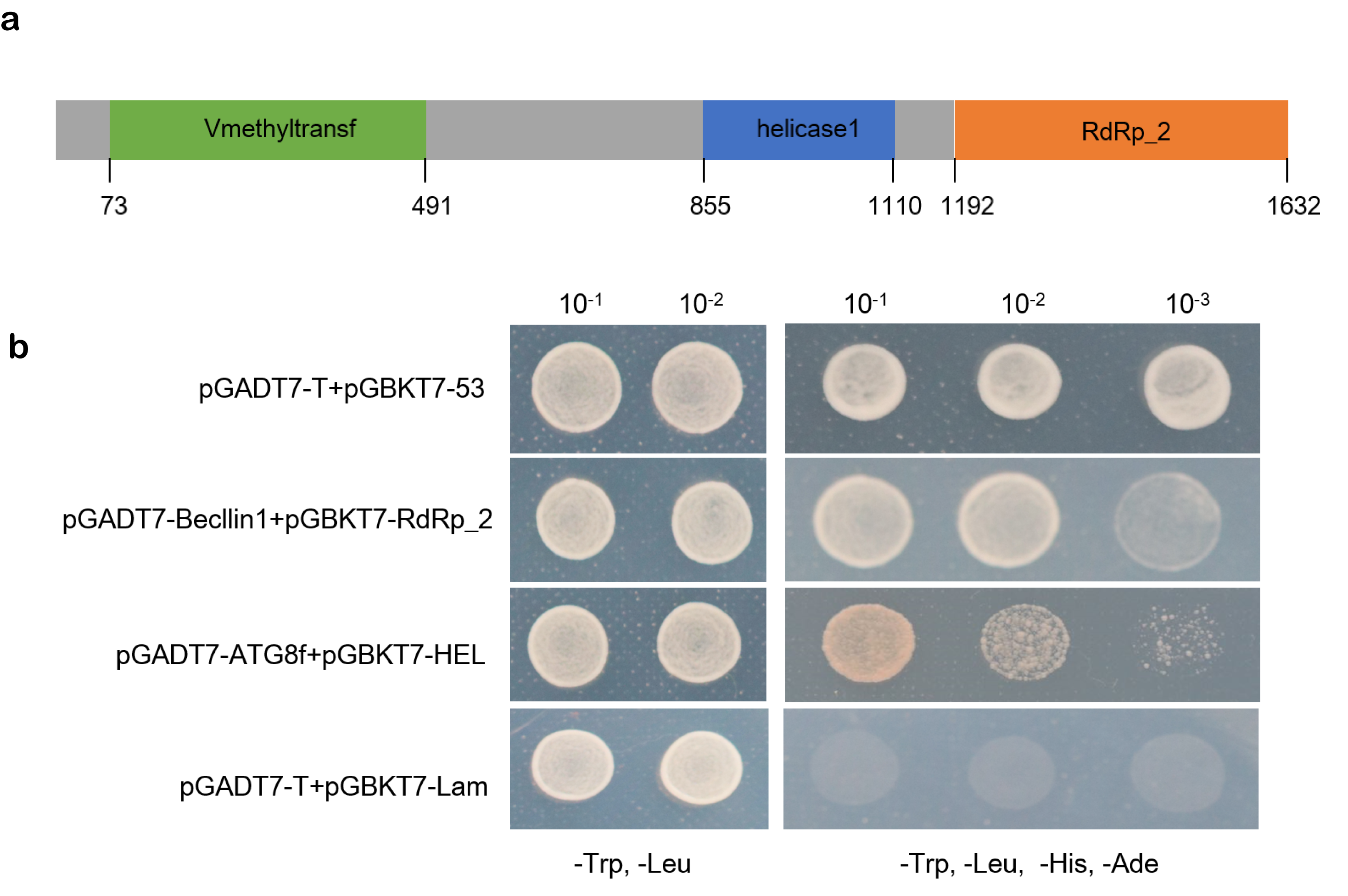

Supplement: Supplementary file 8 — Additional file 8: Figure S3. Analysis of interaction between PMMoV viral proteins and autophagy proteins through yeast two hybrid assay. [file 12870_2020_2711_MOESM8_ESM.tif]

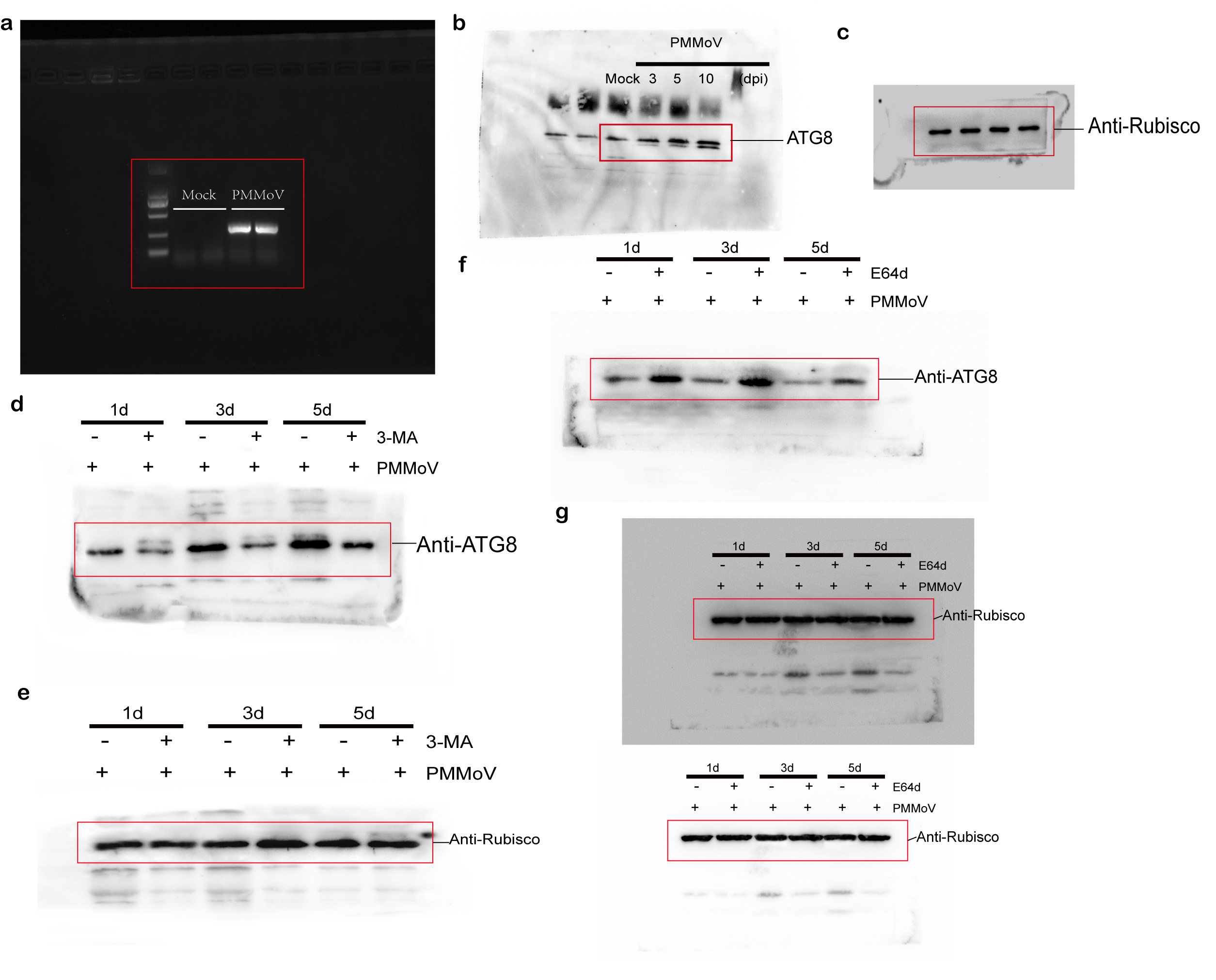

Supplement: Supplementary file 11 — Additional file 11: Figure S4. Source data for Fig. 1b, Fig. 5e and Fig. 6a. (a) Red frame displayed the source data for Fig. 1b. (b-c) Full scan of results shown in Fig. 5e. (d-g) Full scan of the results shown in Fig. 6a. [file 12870_2020_2711_MOESM11_ESM.tif]
